# Long-Term Outcome of Critically Ill Adult Patients with Acute Epiglottitis

**DOI:** 10.1371/journal.pone.0125736

**Published:** 2015-05-06

**Authors:** Tomasz Chroboczek, Martin Cour, Romain Hernu, Thomas Baudry, Julien Bohé, Vincent Piriou, Bernard Allaouchiche, François Disant, Laurent Argaud

**Affiliations:** 1 Service de Réanimation Médicale, Groupement Hospitalier Edouard Herriot, Hospices Civils de Lyon, Lyon, France; 2 Faculté de Médecine Lyon-Est, Université Claude Bernard Lyon 1, Université de Lyon, Lyon, France; 3 Service de Réanimation Médico-chirurgicale, Groupement Hospitalier Lyon-Sud, Hospices Civils de Lyon, Pierre-Bénite, France; 4 Faculté de Médecine Lyon-Sud, Université Claude Bernard Lyon 1, Université de Lyon, Pierre-Bénite, France; 5 Services de Réanimation Chirurgicale, Groupement Hospitalier Edouard Herriot, Hospices Civils de Lyon, Lyon, France; 6 Service d’Oto-Rhino-Laryngologie, Groupement Hospitalier Edouard Herriot, Hospices Civils de Lyon, Lyon, France; University Hospital San Giovanni Battista di Torino, ITALY

## Abstract

**Background:**

Acute epiglottitis is a potentially life threatening disease, with a growing incidence in the adult population. Its long-term outcome after Intensive Care Unit (ICU) hospitalization has rarely been studied.

**Methodology and Principal Findings:**

Thirty-four adult patients admitted for acute epiglottitis were included in this retrospective multicentric study. The mean age was 44±12 years (sex ratio: 5.8). Sixteen patients (47%) had a history of smoking while 8 (24%) had no previous medical history. The average time of disease progression before ICU was 2.6±3.6 days. The main reasons for hospitalization were continuous monitoring (17 cases, 50%) and acute respiratory distress (10 cases, 29%). Microbiological documentation could be made in 9 cases (26%), with *Streptococcus* spp. present in 7 cases (21%). Organ failure at ICU admission occurred in 8 cases (24%). Thirteen patients (38%) required respiratory assistance during ICU stay; 9 (26%) required surgery. Two patients (6%) died following hypoxemic cardiac arrest. Five patients (15%) had sequelae at 1 year. Patients requiring respiratory assistance had a longer duration of symptoms and more frequent anti inflammatory use before ICU admission and sequelae at 1 year (p<0.05 versus non-ventilated patients). After logistic regression analysis, only exposure to anti-inflammatory drugs before admission was independently associated with airway intervention (OR, 4.96; 95% CI, 1.06-23.16).

**Conclusions and Significance:**

The profile of the cases consisted of young smoking men with little comorbidity. *Streptococcus* spp. infection represented the main etiology. Outcome was favorable if early respiratory tract protection could be performed in good conditions. Morbidity and sequelae were greater in patients requiring airway intervention.

## Introduction

Acute epiglottitis is a potentially life threatening disease, especially with severe airway obstruction. Respiratory impairment results from inflammation of the supraglottic structures, usually due to bacterial infection [1,2]. Since the introduction of *Haemophilus influenza* type b (Hib) vaccinations in child immunization programs, the epidemiology of acute epiglottitis has changed, with a significant decline in the incidence among children (and in Hib etiology), and an increase among adults [1–6]. Annual incidence of pediatric epiglottitis in the post-vaccine era has been evaluated as affecting 0.3 to 0.7 per 100,000 patients, with the frequency in adults now greater than in children [2].

The specificities of acute epiglottitis in adult patients in an Intensive Care Unit (ICU) setting have rarely been discussed. Only a few articles focus on adult patients and none, to our knowledge, in the specific setting of the ICU [1,5,7–11]. Moreover, the long-term outcome of the disease remains unknown.

In this paper, we aimed to describe cases of acute epiglottitis in adult patients hospitalized in ICUs, using clinical information recovered at 1 year following the onset of the disease. We also planned to examine factors associated with worst outcome.

## Materials and Methods

### Ethical Considerations

The study received approval from the local ethics committee (*Comité de Protection des Personnes Sud-Est II*). This institutional review board waived the need for consent given the retrospective and non-interventional design of the project. As consent was not obtained, patient records/informations were anonymized and de-identified prior to analysis. This study was conducted in compliance with the amended Declaration of Helsinki and according to French Law.

### Patients

We investigated retrospectively all cases of acute epiglottitis in adult patients *(i*.*e*. *more than 18-year-old)* hospitalized between 1999 and 2013 in 5 ICUs at the teaching hospital in Lyon, France. Each case was defined as swelling of the epiglottis confirmed by direct inspection (fibroscopy) or computerized tomography (CT) scan.

### Data Collection

Clinical, biological, radiological, and therapeutic data were recovered. Comorbidities and the severity of the underlying illness were classified according to the Charlson index and the McCabe scale, respectively [12,13]. Organ failure at admission was categorized using the Sepsis-related Organ Failure Assessment (SOFA) and the Organ Dysfunction and Infection (ODIN) scores [14,15]. Vital status was determined using the Simplified Acute Physiology Score II (SAPS II) [16]. Disease progression was characterized upon discharge from the ICU or from the hospital, or 1 year after onset.

### Statistical Analysis

Results were expressed as mean ± standard deviation (SD) for quantitative variables, and number (percentage) for qualitative data. To establish the factors associated with endotracheal intubation, patient demographics, clinical presentation, diagnostic test results, treatments, and disease progression were compared between ventilated and non-ventilated patients. Univariate comparisons were performed using Fisher's exact test, or chi-squared procedures for categorical data, and independent Student's t-tests for continuous data, as appropriate. All variables with *p* value <0.15 were entered in a backward stepwise multivariate analysis by logistic regression model. Potential confounding factors were eliminated if *p* value was >0.1, but remained in the model if the *p* value was <0.05. We chose to evaluate the impact of at least 1 organ dysfunction rather than organ support or the SOFA score, which were both excluded from the model due to redundancy. Odds ratios (OR) were estimated with 95% confidence intervals (95% CI). Statistical significance was defined as a *p* value less than 0.05.

## Results

Thirty-four cases (sex ratio: 5.8) were investigated. Epidemiological and clinical results are reported in Table 1. Only 8 patients (24%) had no past medical history while 20 (59%) had a history of smoking, alcoholism, or immune depression.

**Table 1:** Patients’ characteristics.

	n = 34
**Epidemiology**	
Male	29 (85)
Age	44 ± 12
**Comorbidities**	
Type	
ENT	10 (29)
Smoking	16 (47)
Alcohol	5 (15)
Immune depression	4 (12)
None	8 (24)
Charlson Index	0.6±1
Life expectancy*	
Non-fatal underlying disease	28 (82)
Ultimately fatal disease (< 5 years)	5 (15)
Rapidly fatal disease (< 1 year)	1 (2.9)
**SAPS II**	24±15
**Length of stay**	
ICU (days)	6±8
Hospital (days)	12±12

ENT: ear nose and throat; SAPS II: Simplified Acute Physiology Score II; ICU: Intensive Care Unit.

* According to McCabe scale

Data are number (%) or mean ± SD, as appropriate.

Data at ICU admission based on the need for airways intervention are presented in Table 2. Average time of disease progression before ICU hospitalization was 2.6±3.6 days. Seventeen patients (50%) had consulted a doctor and gone back home in that same period of time. Of those, 12 (35%) consulted their family doctor, 4 (12%) visited the emergency room, and 1 (0.3%) saw an ear, nose and throat (ENT) specialist. The four most frequent ENT symptoms were throat pain (n = 30, 88%), dysphagia (n = 29, 85%), dyspnea (n = 26, 76%), and dysphonia (n = 22, 65%). The main reasons for ICU hospitalization were the necessity of continuous monitoring (17 cases, 50%), and respiratory distress (10 cases, 29%).

**Table 2:** Patients’ characteristics at admission based on the need for invasive mechanical ventilation.

	Total n = 34	Non-ventilated n = 21	Ventilatedn = 13	*p*
**History before ICU**				
Duration > 48 hours	11 (32)	4 (19)	7 (54)	0.04
Antibiotics prior to hospitalization	9 (26)	5 (24)	4 (31)	0.48
NSAIDs or steroids prior to hospitalization	11 (32)	4 (19)	7 (54)	0.04
**Cause for ICU admission**				<0.0001
Continuous monitoring	17 (50)	15 (71)	2 (15)	-
Respiratory distress	10 (29)	6 (29)	4 (31)	-
Surgery	5 (15)	0	5 (38)	-
Post-respiratory arrest	2 (5.9)	0	2 (15)	-
**Vital signs**				
Temperature	37.3 ± 1.1	37.4 ± 1.1	37.2 ± 1.0	0.69
Mean arterial pressure	102 ± 20	100 ± 22	107 ± 14	0.42
Heart rate	99 ± 22	97 ± 21	103 ± 25	0.51
Spontaneous respiratory rate	19 ± 6	19 ± 6	21 ± 7	0.32
**Biological results**				
White blood cells (G/l)	19.0 ± 7.6	20.7 ± 7.9	16.3 ± 6.6	0.10
C reactive protein (mg/l)	146 ± 126	130 ± 126	188 ± 126	0.28
Bacteriologic respiratory sample	12			
Positive	8 (24)	2 (9.5)	6 (46)	0.001
For *Streptococcus* spp.	7 (21)	2 (9.5)	5 (38)	0.001
Positive blood cultures	1 (2.9)	1 (4.8)	0	0.25
**Organ dysfunction**				
At least 1 dysfunction	8 (24)	0	8 (62)	<0.0001
Type				
Respiratory	8 (24)	0	8 (62)	<0.0001
Cardio-vascular	4 (12)	0	4 (31)	0.01
Renal	3 (9)	0	3 (23)	0.05
Neurologic	2 (5.9)	0	2 (15)	0.14
SOFA score	1.6 ± 3.2	0.3 ± 0.9	3.6 ± 4.4	0.02
**Organ support and treatment**				
Mechanical ventilation	8 (24)	0	8 (62)	<0.0001
Vasopressors	5 (15)	0	5 (38)	<0.01
Surgery	9 (26)	0	9 (69)	<0.0001
Tracheotomy	4 (12)	0	4 (31)	0.01
**Follow-up**				
Hospital mortality	2 (5.9)	0	2 (15)	0.14
1 year mortality	2 (5.9)	0	2 (15)	0.14
1 year sequelae	5 (15)	0	5 (38)	0.01

ICU: Intensive Care Unit; SOFA: Sepsis-related Organ Failure Assessment; NSAIDs: non-steroidal anti-inflammatory drugs.

Data are number (%) or mean ± SD, as appropriate.

Microbiological documentation could be made in 9 cases (26%), by local samples in 8 cases (24%), and by blood tests in the remaining 1 (2.9%). Local samples were positive for *Streptococcus* spp. (n = 7, 21%), including *S*. *anginosus*, (n = 3), β-hemolytic C-group *Streptococcus* (n = 2) and *S*. *viridans* (n = 2); *Fusobacterium necrophorum* (n = 2, 5.9%); *Prevotella* (n = 2, 5.9%), including *P*. *intermedia* (n = 1) and *P*. *oris* (n = 1); *H*. *influenza* (n = 1, 2.9%); *Neisseria* spp. (n = 1, 2.9%); and *Gemella* spp. (n = 1, 2.9%). Multi-bacterial results were obtained in 3 samples (8.8%). The only positive blood culture showed the presence of *H*. *influenza*. CT scans were performed during the disease in 25 patients (74%), showing septic collection, pneumonia, and/or pleural effusion in 13 (38%), 3 (8.8%), and 2 cases (5.9%), respectively. There were no cases of mediastinitis.

Eight patients (24%) presented organ dysfunction on admission, of which all had respiratory failure. Overall, thirteen patients (38%) required respiratory intervention: 6 for surgery (18%), 5 for respiratory failure (15%), and 2 following cardiac arrest (5.9%). Airway management consisted of vigil fibroscopic nasotracheal intubation in 8 cases (24%), orotracheal intubation in 3 cases (8.8%), and emergency tracheotomies in 2 cases (5.9%). Disease duration greater than 48 hours before ICU treatment, use of non-steroidal anti-inflammatory drugs (NSAIDs) or steroids use prior to hospitalization, and positivity for *Streptococcus* spp. were statistically associated with airway intervention (Table 2). After multiple logistic regression analysis, the only variable independently associated with airway intervention was found to be the use of NSAIDs or steroids before admission (OR, 4.96; 95% IC, 1.06–23.16; *p = 0*.*036*).

As the disease progressed, 8 patients (24%) required surgery: 6 (18%) had a collected infection requiring intervention, and 4 (12%) needed tracheotomies. Emergency surgery was performed in 6 cases (18%), and re-interventions occurred in 5 cases (15%). All patients received antibiotics, with ceftriaxone and amoxicillin/clavulanate being the 2 most frequently used (n = 29, 85% and n = 17, 50%, respectively); 18 patients (53%) received a succession of antibiotic treatments, and in 18 cases (53%) were administered a combination of antibiotics. Systemic steroids were given in 22 cases (65%). Adjunctive nebulized therapies with epinephrine, steroids, or hyperbaric oxygenotherapy, were given in 15 (44%), 10 (29%), and 2 cases (5.9%), respectively.

Hospital mortality and mortality at 1 year were similar and concerned only 2 patients (5.9%), who had both presented a cardiac arrest on admission. One year after the onset of the disease, 5 patients (15%) had sequelae: all aesthetic in nature and of which 1 required plastic surgery (2.9%), 3 involved dysphonia (8.9%), and 1 patient (2.9%) had cervical pain. No cases of persistent dyspnea, dysphagia, roaring or stridor were reported. Even if three patients (8.9%) presented another ENT disease during the first year after treatment, no new acute epiglottitis episode was reported.

## Discussion

In this study, we retrospectively investigated 34 cases of acute epiglottitis admitted to the ICU. The profile of the cases consisted of young smoking men, with little comorbidity. *Streptococcus* spp. infection represented the main etiology. Outcome was favorable if early respiratory tract protection could be performed in good conditions. Morbidity and sequelae were heavier in patients requiring airway intervention.

The majority of recent studies (i.e. of the Hib vaccination era) have been carried out in North America, Northern European countries, Australia and East Asia, and have shown that acute epiglottitis is nowadays an adult issue [1–11]. The frequency of childhood epiglottitis was found to be decreasing in the USA, in Canada, Australia, and in Finland [1–3,5,6]. At the same time, the incidence in adults was shown to be growing in the USA, and in Iceland [2–4]. Comparable epidemiological trends have been found in recent literature with the sex ratio rising from 1.1 to 1.6, with the mean age increasing from 40 to 50 years old [1–4,6,7]. We found a highly imbalanced sex ratio, which could mean that cases of acute epiglottitis are more severe in man than in women, with more ICU admissions. Collection bias could also be an explanation. Patients with acute epiglottitis are reported to present medical comorbidities in up to 50% of cases, mainly represented by cardiovascular diseases or cardiovascular risk factors [1–3,7]. This is coherent with our results, with tobacco being a major cardiovascular risk factor. Comorbidity is one of the most important outcome determinants in septic patients [17]. Charlson index and the McCabe scale are commonly used to predict mortality among medical patients, including in case of sepsis, and also in the ICU [12,13,18–22]. Importantly, there was no difference in terms of comorbidities in patients requiring respiratory assistance versus non-ventilated patients.

Microbiological documentation is poorly made in cases of acute epiglottitis [2]. Firstly, blood cultures are rarely contributive since associated bacteremia remains a rare event. Secondly, local swabs are counter-indicated in cases of non-intubated patients with respiratory signs. Finally, reliability of local swabs is questionable. We managed to isolate potentially responsible bacteria in more than a quarter of cases, mainly by local sampling. The most frequent was *Streptococcus* spp., but anaerobic bacteria were found in more than half of cases. *H*. *influenza* was isolated on only two occasions (once in blood test, and once in local swabs). Recent literature reveals poor efficiencies in the isolation of the responsible pathogens, with isolation occurring in 2 to 40% of cases, and rarely with concomitant bacteremia [1,4,7]. When documentation could be made, *H*. *influenzae* appeared to decline drastically after Hib vaccine introduction, and *Streptococcus* spp. to remained the main etiology [1,4]. Based on these results, use of amoxicillin/clavulanate or the association of third generation cephalosporin and metronidazole as a first line empiric therapy, appears adequate [2].

Interestingly, the average duration of symptoms before hospitalization (less than 3 days) was lower in our study than in previous reports [1,2,7]. ICU cases could correspond to a more severe and explosive presentation of acute epiglottitis, explaining these differences. Guardini *et al*. recently found that patients with a more rapid onset of symptoms were more likely to require airway intervention [2]. On the other hand, and as shown previously, we found that a longer duration was associated with a higher probability of intubation, perhaps due to a more severe supraglottic inflammation and obstruction [1]. We also found that the only variable independently associated with airway intervention was the exposure to NSAIDs or steroids before admission. Such medication could have initially tapered respiratory symptoms, extending the time of the disease progression without monitoring, and even worsening the disease before admission. Overall, steroid use remains controversial [2]. Although septic complications can cause heavy morbidity, immediate vital prognosis is related to the protection of the respiratory tract. The two deaths in our study followed hypoxic cardiac arrest. Of the other cases, half had to be ventilated for surgery and the other half for respiratory failure. Fibroscopic nasotracheal intubation was the most frequent technique used. Based on recent studies, up to 60% of patients required ICU hospitalization, and airway intervention was needed in 7 to 21% of cases [1,2,4,7]. When reported, nasal fiber-optic technique remained the most used procedure [2]. Failure in tracheal intubation was reported in up to 14% [2,7]. Although no study has compared different options for airway management, fibroscopic nasotracheal intubation appears to be the most used and recommended technique [1,2]. It presents the advantage of being both diagnostic and therapeutic, but requires the availability of an experienced endoscopist [23]. This should be done in monitored conditions, while maintaining spontaneous breathing in a sitting patient, under supervision of an experienced intensivist, and with concomitant availability of a team capable of immediate tracheotomy [24]. It has previously been reported that if early respiratory tract protection could be performed under good conditions, mortality is low, between 0 to 0.3% [1,2,4,7]. This is similar to our findings. In this situation, respiratory tract protection is an emergency, and no diagnostic procedure should delay it. Of note, extubation should be preceded by a cuff leak test with a deflated cuff [25].

CT scan has already been described as an important step in the management of these patients [26]. It can be performed after intubation or after favorable evolution of respiratory signs. In our study, around 20% of patients required surgery for abscess drainage whereas half of the CT scans disclosed signs of collected infection. This rate is higher than previously published [1,26]. Due to an unpredictable disease progression and unstable respiratory function, unanswered questions remain regarding the timing of imaging studies and management of abscesses.

## Conclusions

In summary, in the present study we found that the majority of patients did not have severity criteria. A few cases presented severe forms of the disease, with vital prognosis threatened by acute airway obstruction. This underlines the need for respiratory screening and early respiratory tract protection. Further studies are needed to outline risk factors associated with airway compromise in adult acute epiglottitis. Long-term sequelae, mainly aesthetic, were also heavier when airway intervention was necessary.
